# Weighted selective collapsing strategy for detecting rare and common variants in genetic association study

**DOI:** 10.1186/1471-2156-13-7

**Published:** 2012-02-06

**Authors:** Yilin Dai, Renfang Jiang, Jianping Dong

**Affiliations:** 1 Department of Mathematical Sciences, Michigan Technological University, Houghton, MI 49931, USA

## Abstract

**Background:**

Genome-wide association studies (GWAS) have been used successfully in detecting associations between common genetic variants and complex diseases. However, common SNPs detected by current GWAS only explain a small proportion of heritable variability. With the development of next-generation sequencing technologies, researchers find more and more evidence to support the role played by rare variants in heritable variability. However, rare and common variants are often studied separately. The objective of this paper is to develop a robust strategy to analyze association between complex traits and genetic regions using both common and rare variants.

**Results:**

We propose a weighted selective collapsing strategy for both candidate gene studies and genome-wide association scans. The strategy considers genetic information from both common and rare variants, selectively collapses all variants in a given region by a forward selection procedure, and uses an adaptive weight to favor more likely causal rare variants. Under this strategy, two tests are proposed. One test denoted by *B_wSC _*is sensitive to the directions of genetic effects, and it separates the deleterious and protective effects into two components. Another denoted by *B_wSCd _*is robust in the directions of genetic effects, and it considers the difference of the two components. In our simulation studies, *B_wSC _*achieves a higher power when the casual variants have the same genetic effect, while *B_wSCd _*is as powerful as several existing tests when a mixed genetic effect exists. Both of the proposed tests work well with and without the existence of genetic effects from common variants.

**Conclusions:**

Two tests using a weighted selective collapsing strategy provide potentially powerful methods for association studies of sequencing data. The tests have a higher power when both common and rare variants contribute to the heritable variability and the effect of common variants is not strong enough to be detected by traditional methods. Our simulation studies have demonstrated a substantially higher power for both tests in all scenarios regardless whether the common SNPs are associated with the trait or not.

## Background

Genome-wide association studies (GWAS) have been used successfully in detecting associations between common genetic variants and complex diseases. However, common SNPs detected by current GWAS only explain a small proportion of heritable variability [1]. These identified common SNPs usually have a relatively small to modest genetic effect, which suggests that another type of variants, rare variants, need to be considered in the current GWAS. Recent studies showed that common diseases can be caused by causal variants with a wide spectrum of allele frequencies including rare alleles [2-4]. In addition to the Common Diseases Common Variants (CDCV) hypothesis underlying complex-disease etiology, an alternative hypothesis, the Common Diseases Rare Variants (CDRV) hypothesis has been the topic of much recent debate [4]. Under this hypothesis, the analysis of accumulative effect of rare variants may become crucial in discovering the link between a candidate gene and the heritable variability missed by the traditional GWAS. There is increasing evidence to support this hypothesis. For example, rare variants associated with type I diabetes hypertension, sterol absorption and plasma levels of LDL have been detected [5-9]. While some studies have shown that rare variants would increase the risk of disease, recent studies also indicate that they could play a 'protective' role for complex traits. For example, multiple rare variants have been shown to act protectively against type I diabetes and hypertension [5,8,9]. With the development of next-generation sequencing technologies, more rare variants can be genotyped so the analysis of association between rare variants and diseases becomes possible. The availability of the sequencing data offers a great opportunity to pursue a very powerful association study considering both common and rare variants. However, the traditional GWAS only adapts for detecting common SNPs. Moreover, it lacks power and requires large sample size for detecting rare variants due to their extremely low allele frequencies. Hence, the development of more powerful statistical tests for association studies using both rare and common variants is needed to meet these challenges.

Recently, a strategy that collapses all rare variants across a causal region was proposed [10]. The idea behind this strategy is to assume that each rare variant in a causal region contributes equally to a disease. Therefore, collapsing genotypes across variants would result in enriched association signals and a reasonably high frequency allele. Several tests based on different collapsing strategies for case-control studies were proposed. One is the Cohort Allelic Sums Test (CAST) [10], in which the numbers of individuals with one or more mutations in a group (e.g. gene) are compared between cases and controls. While CAST only deals with rare variants, the Combined Multivariate Collapsing (CMC) [11] method generalized it by performing a multivariate test with common variants and collapsed scores of rare variants. A weighted sum statistic [12] is another method, which collapses both common and rare variants by adding different weights based on allele frequencies assuming that rare variants have a higher effect than the common ones. One such weighted sum test named ORWSS, whose weights are calculated based on odd ratios, is proposed recently by Feng & Elston and Zhu [13]. Using the regression approaches proposed by Morris & Zeggini [14], those methods can be extended to quantitative phenotypes. Besides the collapsing strategy, several multiple-marker tests have been proposed. Two tests, SSU and SSUw based on sum test have been proposed by Pan [15,16], which can be applied to either common variants or rare variants, but not both. A new adaptive sum strategy proposed by Pan and Shen [17] achieves a selective way to test regions with a few different combinations of genetic variants, which is computationally faster and the result depends on the order of variants. Logistic kernel-machine-based test by Wu [18], which is based on a logistic regression with a kernel function of multiple SNPs, allows for flexible modeling of epistatic and nonlinear SNP effects. The power of a single- marker test is usually low due to the lack of genetic variant information and the need for multiple testing corrections. Multiple-marker tests may also lose power because of higher degrees of freedom. Collapsing methods can avoid drawbacks from both single-marker tests and multiple-marker tests by considering all the genetic variant information with only one degree of freedom.

However, collapsing methods have their own limitations and may not be robust. One limitation is that the classification of rare variants is subjective based on a certain threshold. Tests considering only rare variants cannot utilize genetic information of common variants and lose some power as a consequence. Weighted sum statistics [12,13] were proposed to address this issue by using weights based on minor allele frequencies or log odds ratios. Another limitation is that collapsing methods can be seriously impaired by misclassification of collapsing regions [11]. Regions can usually be defined by genes, SNP allele frequencies, or variant causality. If all rare variants within a collapsing region have the same effect on a disease, for example deleterious effect, the association signal can be amplified; however, if collapsing many non-causal variants, it will introduce noise and adversely affect power. To address this problem, several methods have been proposed recently [19-21]. An adaptive sum test has been proposed [19] to collapse SNPs in a region where their effects have different directions. Each SNP was collapsed positively or negatively based on the marginal association between a trait and itself. Some feature selection based tests [20,21] have also been proposed for rare variants to extract the optimal subset for collapsing by the greedy algorithm strategy such as forward selection and backward elimination. In this article, we develop a weighted selective collapsing method to detect both common and rare variants in a genetic region. We argue that common and rare variants may share a disease risk in the same region. The proposed strategy first selectively collapses common variants into two components representing the deleterious and protective effects by a forward selection procedure according to the correlations. Secondly, using each component as a base, the rare variants are selectively combined into components with a data-driven weight. The final test statistics are developed through a logistic regression model for case control studies.

The proposed strategy tries to consider all information in a genetic region, including both common and rare variants. It addresses the genetic direction problem by using deleterious and protective components and overcomes the issue of non-causal variants by applying a forward selection procedure. To avoid selection bias, a permutation procedure is employed to find the P-value. The method is designed for candidate gene studies of qualitative traits, but it can also be used for genome wide association scan by applying a sliding window strategy and be used for any type of traits through a generalized linear model.

## Results

### Simulation studies

In our simulation studies, we check the type-1 error rate and compare the power of the weighted selective collapsing method (denoted as *B_wSC _*and *B_wSCd_*) with several other tests under various scenarios. The tests are classified into three categories based on genetic resources: rare variants only, common variants only, and both rare and common variants, denoted by R, C, and B, respectively. There are three traditional collapsing methods: the indicator, the sum, and the weighted sum, denoted byind, sum, and wSum, respectively. For example, *R_ind _*represents the test considering only rare variants in a genetic region using an indicator function as collapsing method for all rare variants without any selection. *B_wSum _*is the test using weighted sum collapsing method combining all variants, where the weights are based on minor allele frequencies. The logistic-based single marker test of a common SNP with Bonferroni correction is denoted by *C_bon_*. The logistic-based multiple marker test for common SNPs is denoted by *C_logit_*. Let *B_ind _*and *B_sum _*represent the logistic-based multiple marker tests using all the common SNPs with an extra fake "common SNP", which is obtained by collapsing all rare variants through the indicator and the sum functions as collapsing methods. The selective collapsing method is denoted by SC, and the weighted selective collapsing method is denoted by wSC. The tests which only selectively collapse rare variants are denoted by as $R_{ind}^{SC}$ and $R_{sum}^{SC}$. Let *B_wOR _*be the odds ratio based weighted sum test. *B_SSU _*and *B_SSUw _*are SSU and SSUw tests. *B_aSSU _*and *B_aSSUw _*are both adaptive sum tests using SSU and SSUw as test statistics for all variants. *B_aSSUOrd _*and *B_aSSUwOrd _*are adaptive sum tests for ordered variants. *B_KML _*is Logistic Kernel-Machine Test. Our proposed test are denoted by *B_wSC _*and *B_wSCd_*, which selectively collapse both common and rare SNPs according to the squared correlation coefficients and with data driven weights.

Simulated data are generated based on the strategies used in previous studies [17,22]. A target region with four observed common SNPs and an unobserved causal common SNP in the middle is simulated, while 20 observed non-causal rare SNPs and 8 causal rare SNPs are also simulated independently with common SNPs. For each sample, common SNPs are generated based on a latent variable *Z *= (*Z*_1_, . . . , *Z*_5_)' from a multivariate normal distribution with covariance structure *Corr*(*Z_i_*, *Z_j_*) = 0.4 between any two observed components. Each observed common SNP has the same chance to correlate with the underlying causal SNP with *Corr*(*Z_i_*, *Z*_3_) = a * 0.4, where a takes values 1 and -1 with probability 0.5. Each allele on the haplotype is generated with a minor allele frequency obtained from a uniform distribution between 0.1 and 0.3. Rare variants are generated independently with common SNPs, which are also from a multivariate normal distribution. Within each group of no causal rare variants and causal rare variants, LD structure is defined by *Corr*(*Z_i_*, *Z_j_*) = 0.4^|*i*-*j*|^. Each allele on a haplotype is generated with the cut-off of the minor allele frequency obtained from a uniform distribution between 0.001 and 0.005. Next, genotypes *X_i _*= (*X*_*i*1_, . . . , *X*_*i*32_)' for each individual are generated by the sum of two haplotypes. Last, the phenotype Y_i _is generated based on the logistic regression model with a given odds ratio and the order of genotypes have been shuffled. We consider five scenarios here. Scenario A is the null case where the odds ratios for all variants are set at 1. In Scenario B, rare variants are associated with the trait but common variants do not. We randomly selected eight with the customized odd ratio by parameter, *OR *between 1.3 and 3.1. Odd ratio of the half rare variants is defined as *OR *and another half is defined as *OR *plus one. For example, if *OR *is 2, then we consider *Odds Ratio *= (2, 2, 2, 2, 3, 3, 3, 3) for eight casual rare variants. In Scenario C, both common and rare variants have effects on the traits, but effects from common variants are not significant enough to be detected by traditional association approaches. The odds ratio of the unobserved causal common SNP is set at 1.5. The odds ratios for rare variants are set in the same fashion as in Scenario B. Scenario D, which is quite similar to Scenario B, has a different odds ratio structure for rare variants. The odds ratio for half of them is set to be positive, while it is set to be negative for the rest. For example, if *OR *is 2, then we consider $Odds\;Ratio=\left(2,2,\frac{1}{2},\frac{1}{2},3,3,\frac{1}{3},\frac{1}{3}\right)$ for eight casual rare variants to reflect possible different genetic effect. Scenario E is the counterpart version of Scenario C considering odds ratios to reflect possible different directions. 500 cases and 500 controls are simulated in the study with 1000 simulation replicates and the significant level was set at 0.05 for all scenarios.

### Type-I error rate and Power

For tests requiring a permutation procedure, a quicker way for calculating P*- *values is to simulate a large sample of test statistics from the asymptotic null distribution. We randomly select 1,000 simulation replicates and shuffle the phenotype data 1,000 times to generate data under the null hypotheses and compute the tests statistics for the asymptotic null distribution. We first consider Scenario A to check the type-I error rate. In Table 1 we can see that all tests have satisfactory Type I error rates.

**Table 1:** Type I error rates for all tests in simulated data of scenario A

Test	Type-1 error	Test	Type-1 error	Test	Type-1 error	Test	Type-1 error
*R_ind_*	0.054	$R_{ind}^{SC}$	0.051	*B_SSU_*	0.053	*B_aSSUOrd_*	0.06

*R_sum_*	0.053	$R_{sum}^{SC}$	0.054	*B_SSUw_*	0.042	*B_aSSUwOrd_*	0.062

*C_bon_*	0.054	*B_ind_*	0.055	*B_aSSU_*	0.062	*B_wSC_*	0.042

*C_logit_*	0.055	*B_sum_*	0.058	*B_aSSUw_*	0.055	*B_wSCd_*	0.051

*B_wSum_*	0.055	*B_wOR_*	0.062	*B_KML_*	0.056		

There is customized LD structure among common variants and among rare variants. *R_ind_*, collapsing method by indicator function on rare variants. *R_sum_*, collapsing method by sum function on rare variants. $R_{ind}^{SC}$, selective *R_ind_*. $R_{sum}^{SC}$ selective *R_sum _*. *C_bon_*, single test with bonferroni correction on common variants. *C_logit_*, multivariate logistic regression test on common variants. *B_ind _*and *B_sum_*, *C_logit _*with collapsed component from rare variants. *B_wSum_*, weighted sum test. *B_wOR _*Odds Ratio based weighted sum test. *B_SSU_*, *B_SSUw _*, *SSU *based tests. *B_aSSU_*, *B_aSSUw_*, adaptive sum tests. *B_aSSUOrd_*, *B_aSSUwOrd _*ordered adaptive sum tests. *B_KML_*, Logistic Kernel-Machine Test. *B_wSC_*, *B_wSCd _*selectively weighted collapsing.

Under the alternative hypothesis, we first consider the case where all rare variants have the same genetic effect on the trait. In scenario B, where only rare variants are associated with the trait, we consider tests R and B, a total of 17 tests. The result is shown in Table 2. The proposed test *B_wSC _*achieves the highest power under different OR. Roughly speaking, *B_wSC_*, *B_wSum_*, $R_{sum}^{SC}$, $R_{ind}^{SC}$ and *B_aSSUwOrd _*are the top five tests among 17 tests. Multivariate tests with common variants and an extra component from rare variants, *B_ind _*and *B_sum_*, have low power as expected, because common variants do not contribute to the trait variability so they are just noise. *R_sum _*has a consistently better performance than R_ind_. However, among all variants, more than half of them are non-causal, which are also noise in this case. Directly collapsing without any selection would lead to a loss of power. $R_{ind}^{SC}$ and $R_{sum}^{SC}$ achieve a relative higher power than *R_ind _*and *R_sum _*by a selection procedure to remove the noise from the non-causal rare variants. *B_wSum_*, on the other hand, puts more weight on the rare variants to reduce noise in this scenario, resulting a better performance than previous tests. However, as shown in the appendix, the weights based on the estimated minor allele frequencies from controls tend to favor those deleterious rare variants and to ignore the protective rare variants. Thus, scenario B, where all causal variants are deleterious, is the optimal case for *B_wSum_*. *B_wSC _*achieves the highest power by considering both common and rare variants with a selection procedure and a data driven weight which could benefit both deleterious and protective rare variants and reduce noise. *B_wOR _*has a lower power in this simulation study, because, for a region with the limited number of variants, we used the weights from log odds ratios without additional threshold. This may not be significantly enough to distinguish the true signal and noise. In this simulation study, the order of all variants is shuffled to have a fair comparison with adaptive tests. *B_aSSUwOrd _*achieves a higher power by sorting the genotypes according to single test statistics and performs an adaptive SSUw test. *B_aSSUwOrd _*has a consistently better performance than *B_SSUw _*and *B_aSSUw _*in both cases. SSUw based tests have a consistently better performance than SSU based tests.

**Table 2:** Power for all tests in simulated data of scenario B, no common SNPs effect, effects of RVs are in the same directions

*OR*	1.3	1.6	1.9	2.2	2.5	2.8	3.1
*R_ind_*	0.227	0.376	0.522	0.63	0.737	0.81	0.851

*R_sum_*	0.245	0.424	0.57	0.67	0.778	0.846	0.888

*B_ind_*	0.129	0.204	0.318	0.419	0.522	0.623	0.698

*B_sum_*	0.147	0.243	0.343	0.47	0.565	0.674	0.751

$R_{ind}^{SC}$	0.295	0.42	0.589	0.726	0.834	0.884	0.954

$R_{sum}^{SC}$	0.298	0.425	0.588	0.731	0.834	0.894	0.946

*B_wSum_*	0.302	0.474	0.631	0.71	0.81	0.875	0.931

*B_wOR_*	0.09	0.17	0.226	0.295	0.416	0.408	0.58

*B_KML_*	0.044	0.054	0.057	0.067	0.08	0.074	0.078

*B_SSU_*	0.042	0.049	0.053	0.062	0.075	0.071	0.07

*B_SSUw_*	0.136	0.257	0.386	0.592	0.706	0.814	0.866

*B_aSSU_*	0.074	0.106	0.197	0.219	0.275	0.324	0.351

*B_aSSUw_*	0.161	0.243	0.378	0.504	0.691	0.755	0.823

*B_aSSUOrd_*	0.234	0.325	0.468	0.628	0.738	0.849	0.877

*B_aSSUwOrd_*	0.211	0.293	0.462	0.629	0.793	0.847	0.896

*B_wSCd_*	0.201	0.34	0.445	0.586	0.734	0.825	0.885

*B_wSC_*	0.316	0.509	0.654	0.775	0.892	0.927	0.97

There is a customized LD structure among common variants and among rare variants.

Randomly selected eight rare variants are casual variants. Others are non- casual variants. Genetic effect parameter *OR *for eight rare variants is listed in the table. If *OR *is 2, *Odds Ratio *= (2, 2, 2,3, 3, 3) for eight casual rare variants. Notations of tests are defined similarly those in Table 1.

**Figure 1:**
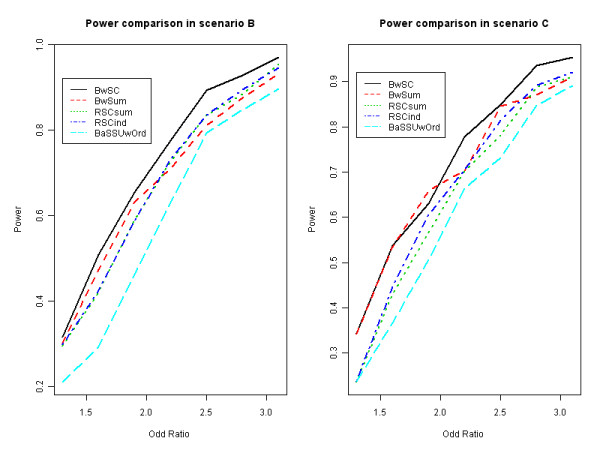
**Power comparison in scenarios B and C**. Selected tests are considered for power comparison in scenarios B and C, where all causal rare variants have the same genetic effect on the trait. Scenario B is the case that only rare variants affect the traits, while Scenario C is the case that both common and rare variants affect the traits. RSCsum represents selective R_sum_. RSCind represents selective R_ind_. BwSum represents weighted sum test. BaSSUwOrd represents ordered adaptive sum test with test statistics of SSUw. BwSC represents weighted selectively collapsing test sensitive to the direction.

When the effect of rare variants is relatively weak (OR is from 1.3 to 2.2), *R_ind _*and *R_sum _*perform better than *B_aSSUwOrd_*. *B_KML _*and *B_SSU _*have the lowest power in this simulation study. *B_KML _*has a consistently better performance than *B_SSU_*. In scenario C, both common and rare variants are associated with the trait, but the association between common SNPs and the trait is not strong enough to be detected by the traditional association methods. We considered all 19 tests, the results are shown in Table 3. Our test, *B_wSC_*, achieves the highest power in most case of OR, except when OR = 1.9, *B_wSum _*has a slight higher power. Roughly speaking, *B_wSC _B_wSum_*, $R_{sum}^{SC}$, $R_{ind}^{SC}$ and *B_aSSUwOrd _*are the top five among 19 tests. *B_KML _*and *B_SSU_*, either using a linear kernel or without using any weights on rare variants, result in the same power as *C_bon _*and *C_logit _*in this simulation study. The results of selected tests in scenario B and C, where all rare variants have the same genetic effect on the trait, are shown in Figure 1 to demonstrate the comparison.

**Table 3:** Power for all tests in simulated data of scenario C weak common SNPs effect, effects of RVs are in the same direction

*OR*	1.3	1.6	1.9	2.2	2.5	2.8	3.1
*R_ind_*	0.237	0.394	0.472	0.6	0.715	0.785	0.843

*R_sum_*	0.247	0.418	0.543	0.636	0.747	0.811	0.869

*C_bon_*	0.163	0.157	0.144	0.164	0.174	0.191	0.193

*C_logit_*	0.195	0.199	0.193	0.207	0.212	0.228	0.238

*B_ind_*	0.278	0.364	0.436	0.517	0.618	0.677	0.76

*B_sum_*	0.298	0.384	0.461	0.562	0.668	0.735	0.795

$R_{ind}^{SC}$	0.236	0.43	0.565	0.702	0.781	0.888	0.91

$R_{sum}^{SC}$	0.238	0.446	0.605	0.705	0.815	0.892	0.92

*B_wSum_*	0.341	0.534	0.658	0.703	0.846	0.87	0.911

*B_wOR_*	0.253	0.312	0.344	0.475	0.456	0.582	0.648

*B_KML_*	0.167	0.186	0.186	0.19	0.204	0.199	0.2

*B_SSU_*	0.165	0.179	0.179	0.181	0.192	0.192	0.188

*B_SSUw_*	0.203	0.334	0.458	0.61	0.716	0.808	0.861

*B_aSSU_*	0.168	0.215	0.235	0.28	0.303	0.34	0.383

*B_aSSUw_*	0.181	0.346	0.399	0.546	0.64	0.755	0.819

*B_aSSUOrd_*	0.163	0.293	0.376	0.571	0.592	0.733	0.798

*B_aSSUwOrd_*	0.238	0.367	0.506	0.663	0.732	0.847	0.89

*B_wSCd_*	0.21	0.395	0.484	0.625	0.661	0.822	0.848

*B_wSC_*	0.344	0.538	0.631	0.778	0.85	0.935	0.954

There is customized LD structure among common variants and among rare variants.

The *OR *for underlying common SNP is 1.5. Genetic effect parameter *OR *for eight rare variants is listed in the table. If *OR *is 2, *Odds Ratio *= (2, 2, 2, 2, 3, 3, 3, 3) for eight casual rare variants. Notations of tests are defined similarly as those in Table 1.

**Figure 2:**
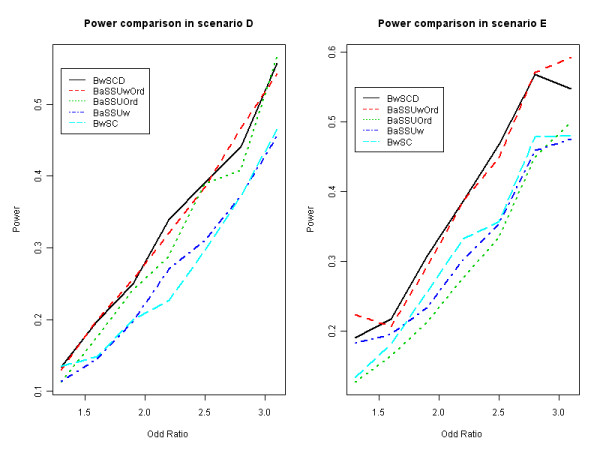
**Power comparison in scenarios D and E**. Selected tests are considered for power comparison in scenarios D and E, where causal rare variants have different genetic effect on the trait. Scenario D is the case where only rare variants affect the traits, while Scenario E is the case where both common and rare variants affect the traits. BwSCd represents weighted selectively collapsing test robust in the direction. BaSSUOrd represents ordered adaptive sum test with test statistics of SSU. BaSSUw represent adaptive sum test with test statistics of SSUw. Other names of tests are defined similarly as in Figure 1.

Now, we consider scenarios D and E where rare variants have different genetic effect on the trait. Tables 4 and 5 show the results of these two scenarios. *B_wSCd_*, achieves the highest power in scenario D for most case of OR. When OR = 1.9 and 2.8, *B_aSSUwOrd _*achieves the highest power. When OR = 3.1, *B_aSSUOrd _*achieves the highest power. Roughly speaking, *B_wSCd_*, *B_aSSUwOrd_*, *B_aSSUOrd_*, *B_aSSUw _*and *B_wSC _*are the top five tests among 17 tests in scenario D. In scenario E, *B_wSCd _*and *B_aSSUwOrd _*achieve the highest power in most cases. When OR = 1.3 and 1.6, *B_wOR _*achieves the highest power. When OR = 1.3, 2.8 and 3.1, *B_aSSUwOrd _*has a higher power than *B_wSCd_*. When OR = 1.6, 1.9 and 2.5, *B_wSCd _*has a higher power. When OR = 2.2, they both achieve the same power. Roughly speaking, *B_wSCd_*, *B_aSSUwOrd_*, *B_wSC_*, *B_aSSUw _*and *B_SSUw _*are the top five test among 19 tests. Being different from the results of scenarios B and C, the power of *B_wSum _*drops significantly, because the weights in *B_wSum _*only favor those deleterious rare variants and ignore the protective rare variants, which are as important as deleterious ones in this simulation. Although *B_wOR _*achieves a low power because of limit number of variants, *B_wOR _*has performed consistently better than *B_wSum _*in most cases under both scenarios. Due to the presence of the causal rare variants with opposite association directions and non-causal rare variants, other tests involving directly collapsing methods also have a lower power. On the other hand, SSU and SSUw based tests tend to perform well under these scenarios. *B_aSSUwOrd _*becomes one of the most powerful test in these two scenarios. We find that SSUw based tests combine both deleterious and protective genetic variations into the test statistic SSUw, while most collapsing methods only consider one of them. Having the same merit of *B_aSSUwOrd_*, our second proposed method *B_wSCd_*, which is based on the difference of the two components, achieves the higher power in most cases. The results of selected tests in scenario D and E, where rare variants have different genetic effect on the trait, are shown in Figure 2.

**Table 4:** Power for all tests in simulated data of scenario D, no common SNPs effect, effects of RVs are in the different directions

*OR*	1.3	1.6	1.9	2.2	2.5	2.8	3.1
*R_ind_*	0.062	0.058	0.089	0.095	0.118	0.129	0.164

*R_sum_*	0.054	0.062	0.092	0.083	0.113	0.118	0.158

*B_ind_*	0.062	0.06	0.059	0.074	0.085	0.1	0.128

*B_sum_*	0.062	0.059	0.065	0.073	0.09	0.101	0.117

$R_{ind}^{SC}$	0.09	0.15	0.214	0.221	0.314	0.352	0.395

$R_{sum}^{SC}$	0.094	0.151	0.202	0.21	0.335	0.353	0.449

*B_wSum_*	0.107	0.096	0.096	0.136	0.179	0.221	0.27

*B_wOR_*	0.09	0.126	0.133	0.165	0.211	0.222	0.255

*B_KML_*	0.061	0.055	0.054	0.054	0.067	0.067	0.072

*B_SSU_*	0.056	0.053	0.052	0.05	0.062	0.062	0.068

*B_SSUw_*	0.095	0.126	0.181	0.254	0.314	0.354	0.478

*B_aSSU_*	0.086	0.087	0.13	0.138	0.167	0.162	0.229

*B_aSSUw_*	0.114	0.145	0.198	0.271	0.311	0.373	0.456

*B_aSSUOrd_*	0.113	0.175	0.241	0.289	0.39	0.409	0.566

*B_aSSUwOrd_*	0.129	0.2	0.256	0.321	0.385	0.468	0.543

*B_wSC_*	0.135	0.148	0.2	0.227	0.297	0.373	0.465

*B_wSCd_*	0.134	0.197	0.25	0.34	0.391	0.441	0.558

There is a customized LD structure among common variants and among rare variants.

Randomly selected eight rare variants are causal variants. Others are non-causal variants. Genetic effect parameters *OR *for eight rare variants are listed in the table. Odds Ratios for another half of rare variants are in different directions. If *OR *is 2, $Odds\;Ratio=\left(2,2,\frac{1}{2},\frac{1}{2},3,3,\frac{1}{3},\frac{1}{3}\right)$ for eight casual rare variants. Notations of tests are defined similarly as those in Table 1.

**Table 5:** Power for all tests in simulated data of scenario E, weak common SNPs effect, effects of RVs are in the different directions.

*OR*	1.3	1.6	1.9	2.2	2.5	2.8	3.1
*R_ind_*	0.045	0.077	0.068	0.103	0.115	0.12	0.157

*R_sum_*	0.054	0.074	0.062	0.091	0.109	0.126	0.154

*C_bon_*	0.156	0.131	0.155	0.139	0.186	0.149	0.146

*C_logit_*	0.211	0.185	0.214	0.192	0.221	0.211	0.19

*B_ind_*	0.2	0.184	0.2	0.198	0.244	0.225	0.233

*B_sum_*	0.19	0.182	0.2	0.197	0.243	0.226	0.229

$R_{ind}^{SC}$	0.068	0.122	0.176	0.241	0.27	0.359	0.387

$R_{sum}^{SC}$	0.094	0.119	0.193	0.254	0.273	0.371	0.39

*B_wSum_*	0.1	0.114	0.164	0.172	0.193	0.236	0.272

*B_wOR_*	0.201	0.245	0.26	0.311	0.334	0.398	0.405

*B_KML_*	0.169	0.167	0.186	0.159	0.197	0.173	0.171

*B_SSU_*	0.166	0.161	0.175	0.153	0.189	0.167	0.163

*B_SSUw_*	0.146	0.169	0.241	0.306	0.395	0.445	0.521

*B_aSSU_*	0.139	0.148	0.196	0.185	0.212	0.256	0.255

*B_aSSUw_*	0.183	0.196	0.233	0.302	0.354	0.459	0.476

*B_aSSUOrd_*	0.127	0.164	0.213	0.276	0.334	0.45	0.5

*B_aSSUwOrd_*	0.224	0.206	0.293	0.386	0.449	0.571	0.593

*B_wSC_*	0.133	0.182	0.256	0.332	0.357	0.479	0.48

*B_wSCd_*	0.19	0.217	0.308	0.386	0.468	0.568	0.548

There is a customized LD structure among common variants and among rare variants. The OR for underlying common SNP is 1.5. Randomly selected eight rare variants are causal variants. Others are non-causal variants. Genetic effect parameters *OR *for eight rare variants are listed in the table. Odds Ratios for half of rare variants are in different directions. If *OR *is 2, $Odds\;Ratio=\left(2,2,\frac{1}{2},\frac{1}{2},3,3,\frac{1}{3},\frac{1}{3}\right)$ for eight casual rare variants. Notations of tests are defined similarly as those in Table 1.

## Discussion

In this paper, we proposed two novel association tests for candidate gene studies and genome wide association studies. The test *B_wSC _*selectively collapses common and rare variants into two separate components with data-driven weights. The test statistic is derived by comparing these components, which is robust in situations with or without common variants. A permutation procedure is employed to find the P-value. Simulation studies show that the proposed tests achieve a higher power than other commonly used tests for rare variants in most cases. The optimal scenario for the proposed test is that when the common and rare variants both contribute to the heritable variability and effects of common variants are not detectable by traditional methods using common variants alone. If there is no association between the common variants and the trait, the proposed method also performs robustly as well as demonstrated by our simulation studies. We believe that the improved power comes from three sources. First, the test considers more genetic information by combining both common and rare variants instead of dealing with rare variants alone. Second, the test filters out the suspicious non-causal variants as noise and separates the variants into deleterious ones and protective ones by the selective collapsing method. Distinguishing deleterious and protective sources can improve the power when variants have different genetic effect on the trait. For example, in the worst case scenario, common variants have a deleterious effect, while rare variants collectively have a protective effect on the trait. The effects from the two sources will be neutralized if the effect directions are not distinguished. Our test can achieve a high power by choosing the strongest source in any cases instead of neutralizing them. The third reason for the improvement of the power comes from the data driven weights. Instead of using weights based on estimates of the minor allele frequencies from control data, which favor those deleterious rare variants and ignore the protective rare variants, the proposed test uses weights based on an estimate of the disease risk, which is the probability of an individual with disease mutation. The proposed weights tend to favor both deleterious and protective rare variants.

Although the proposed test (*B_wSC_*) has many advantages, it is certainly not universally better than other tests. For example, in scenarios D and E, when the mixed genetic effect exists, *B_wSC _*can only capture the genetic effect in one direction. It can be used for detecting variants with the same genetic effect direction. Therefore, we also propose another test *B_wSCd_*, which can capture all genetic effect. It can be used for detecting a region of variants with opposite directions of genetic effects. We also would like to point out that the proposed test can be easily extended to include covariates since the tests are based on a logistic regression model. It can also be applied to quantitative traits by using a linear regression model. The strategy that collapsing rare variants based on common variants for qualitative trait in GWAS has been successfully applied to the simulated sequencing data from Genetic Analysis Workshop 17[23], where a GWAS permutation procedure of our method was proposed for qualitative trait as well.

## Conclusions

In summary, we proposed two weighted selectively collapsing tests for both candidate gene studies and genome-wide association studies; in the latter case, the analysis unit can be based on genes, pathways, or sliding windows. The two tests are potentially powerful methods for association studies in sequencing data by combining all variants information, by filtering out suspicious non-causal variants, and by using adaptive weight on likely causal rare variants. One test is robust in the directions of genetic effects, and it adapts to the region with mixed genetic effects. Another test is sensitive to the directions of genetic effects, and it adapts to the region with same genetic effect. It is designed mainly for detecting rare variants, and it achieves a higher power by considering common variants when needed. Our simulation studies have demonstrated their substantially higher power in all scenarios by combining advantages from other existing tests.

## Method

We focus on qualitative traits only in this study. It can be easily extended to any other traits through a generalized linear model. Different variants and collapsing strategies are considered within the framework of logistic regression. We also compared some recently proposed methods, SSU tests[15], adaptive tests [17], ORWSS [13] and Logistic Kernel-Machine Test[18] in our simulation study. The goal of this work is to detect any association between the trait and a given genetic region which includes both common and rare variants. Consider an association study with *N *samples in a genetic region with *K *variants. Let *Y_i _*denote the coded trait for the *ith *sample, 0 for controls and 1 for cases. The variants were coded by an additive genetic model: *X_ik _*was coded as 0, 1, and 2 as genotype scores for the *kth *marker of the *ith *sample, where *i *= 1, . . . , *N*, and *k *= 1, . . . , *K*. Let $X_{ik}^{C}$ and $X_{ik}^{R}$ be common variants and rare variants based on a certain threshold. For example, SNPs with minor allele frequencies less than 0.01 are considered as rare variants.

### Collapsing Methods and Logistic Regression

Collapsing approaches have been previously proposed using either an indicator function or a sum (proportion) function [11,14]. Let S_i _denote the collapsed score for a genetic region. The indicator function based collapsing method is $S_{i}=I\left(\sum_{k=1}^{K}X_{ik}^{R}\right)$ and the sum (proportion) function based collapsing method is $S_{i}=\sum_{k=1}^{K}X_{ik}^{R}$.

In a case control study, it is natural to consider the logistic regression model for tests, and those collapsing methods can be achieved by: Logit Pr(*Y_i _*= 1) = *β*_0_, + *β*_1_*S_i_*. The null hypothesis of no genetic effect is H_0 _: β_1 _= 0. In a candidate gene study, we employed the likelihood ratio test. Because the score test is computationally faster than the likelihood ratio test, we use the following tests for the genome wide association study. Let

$$U=\overset{N}{\underset{i=1}{\sum }}\left(Y_{i}-Ȳ\right)S_{i}$$

and

$$V=Ȳ\left(1-Ȳ\right)\overset{N}{\underset{i=1}{\sum }}{\left(S_{i}-\bar{S}\right)}^{2}$$

where $Ȳ=\frac{\Sigma_{i=1}^{N}Y_{i}}{N}$ and $\bar{S}=\frac{\Sigma_{i=1}^{N}S_{i}}{N}$.

The score test is

$$T_{s}=\frac{U^{2}}{V}$$

which has an asymptotic χ^2 ^distribution with degrees of freedom one.

The limitation of the current collapsing approaches is that they only consider rare variants. For example, when common variants contribute to the heritable variability not detectable by the traditional common SNPs approaches, ignoring them will lose power of the tests.

The Combined Multivariate Collapsing method (CMC) [11] solves this problem by regarding collapsed score as a common SNP and performing a Hotelling's *T*^2 ^test on multiple markers. To put this method within our logistic regression framework, we consider a multivariate logistic regression model.

$$\text{Logit}\;\text{Pr}\left(Y_{i}=1\right)=\beta_{0}+\beta_{1}S_{i}+\underset{k}{\sum }\beta_{k}^{c}X_{ik}^{c}$$

The null hypothesis of no genetic effect is ${\text{H}}_{0}:\beta_{1}=\beta_{\text{k}}^{\text{c}}=0$.

Another collapsing method uses a data-driven weight considering both common and rare variants.

$$S_{i}=\overset{K}{\underset{k=1}{\sum }}w_{k}X_{ik}$$

where the weight is calculated by $w_{k}=\frac{1}{\sqrt{{\hat{q}}_{k}\left(1-{\hat{q}}_{k}\right)}}$, ${\hat{q}}_{k}=\frac{\sum_{i\in control}X_{ik}}{2N_{0}+2}$ and *N*_0 _is the number of controls in the study [12]. By using a weight, the collapsed score amplifies the contribution of rare variants. The test statistic can be derived from logistic regression as before. Because the weights are data-dependent, a permutation test is employed to find P-values.

For a region with both common and rare variants, the above two approaches consider all the genetic information. However, it is impossible that all variants in this region contribute to the heritable variability, and it is more likely that only some of them are causal. If many of rare variants are non-casual, collapsing will inevitably introduce noise and lose power of the test.

A covering method called RareCover [21], has been recently proposed to determine a collapsing subset from all the variants in this region using a forward selection procedure. For the purpose of comparison, we also put this strategy in our logistic regression framework. Instead of using Pearson's χ^2^, which was used by the original authors, we considered the squared correlation coefficient R^2 ^as the screening test statistic. Starting from a score without any rare variants, each rare variant is examined, and it is added into this score if it improves the test statistic the most. An optimal subset was obtained by a forward selection procedure to achieve the highest squared correlation between the collapsed score and traits. The test statistic then can be derived from a logistic regression model between the trait and the collapsed score as before. P-value can be found by permutation. However, this method does not consider genetic information from the common variants in this region and it ignores the direction of the rare variants by using either the squared correlation coefficient *R*^2 ^or Pearson's *χ*^2^.

### Recent proposed multi-marker tests

We also compared some recently proposed methods, SSU tests[15], adaptive tests [17], ORWSS [13] and Logistic Kernel-Machine Test[18] in our simulation studies. We briefly review these methods here. SSU and SSUw tests are defined as follow.

Let the score vector *U *= (*U*_1_, . . . , *U_K_*), where each component $U_{k}=\sum_{i=1}^{N}X_{ik}\left(Y_{i}-Ȳ\right)$, and $\bar{\text{Y}}$ are the sample mean of phenotype.

*SSU *= *U*'*U *And *SSUw *= *U*'*Diag*(*If*)^-1^*U*, Where *I_f _*= *Cov*(*U*) is the expected fisher information matrix. Asymptotic distributions of the above two test statistics are scaled *χ*^2 ^distributions[15].

For the Adaptive test, suppose that *U_m _*= (*U*_1_, . . . , *U_m_*), where *m<K*, is the vector containing the first m components. Adaptive test statistics is

$$aT=min_{1\leq m\leq K}Pval\left(T\left(U_{m}\right)\right)$$

where *Pval*(*T*(*U_m_*)) is the p-value of the test statistic, T. For the Adaptive test, we used SSU and SSUw as the score of the test statistics T. The adaptive tests are called aSSU and aSSUw tests. More generally, one can order the SNPs based on the single test statistics and repeat the adaptive test process, resulting in the aSSU-Ord and aSSUw-Ord. The P-value of *aT *is calculated by a permutation procedure.

For the ORWSS test, the score is constructed in the same way as other weighted sum test.

$$S_{i}=\overset{K}{\underset{k=1}{\sum }}w_{k}X_{ik}$$

but the weight is calculated as follow.

The amended estimator of the odds ratio is computed by adding 0.5 to each cell of the 2 by 2 table for case control studies. If we define *γ_k _*= *log*(*OR_k_*), where *OR_k _*is the odds ratio for the k*th *marker.

$$w_{k}=\left\{\begin{array}{cc} \begin{array}{c} \gamma_{k}\\ 0 \end{array} & \begin{array}{c} if|\gamma_{k}-\bar{\gamma_{k}}|>c\sigma \\ otherwise \end{array} \end{array}\right)$$

where *σ *is the standard deviation calculated from γ_k_, k = 1, . . . , K, c is a parameter and $\bar{\gamma_{\text{k}}}$ is the mean of log odds ratios[13]. In the simulation study, because number of variants is small, we using the logarithm of odds as a weight directly for each SNP without classification.

Then the test statistic is defined as

$$\text{ORWSS}=\underset{i\in Case}{\sum }rank\left(S_{i}\right)$$

P-value of ORWSS is calculated by a permutation procedure.

For the Logistic Kernel-Machine Test, the test statistics is based on logistic regression with a kernel function of the SNPs.

$$\text{Logit}\;\text{Pr(}Y_{i}=1\text{)}=\beta_{0}+h\left(X_{i1},\cdots \;,X_{iK}\right)$$

Some commonly used kernels include linear, identity-by-descent (IBS) and quadratic kernels. We only consider the linear kernel here. In order to test whether there is a true genetic effect, the null hypothesis is H_0 _: *h*(*X*) = 0. The test statistics has been developed as

$$\text{Q}=\frac{{\left(Y-Ȳ\right)}^{\prime }K\left(Y-Ȳ\right)}{2}$$

which follows a scaled *χ*^2 ^distribution[18].

For all the tests above, we considered both common and rare variants, since we want to develop a robust strategy to detect any association between complex traits and genetic regions considering both common and rare variants.

### Weighted Selective Collapsing Strategy

Now, we propose a new collapsing strategy, which considers genetic information from both common and rare variants. The new strategy tries to remove the noise generated by the non-causal variants and to improve the power by considering both deleterious and protective components of this region. In brief, our strategy is as follows. We defined rare variants as SNPs with minor allele frequencies less than 0.01, others as common variants. Starting from a null model without any variants, by a forward selection procedure, common SNPs are first selectively collapsed into two components, which will serve as bases for the rare variants. One is a deleterious component having an extremely positive correlation coefficient with the trait. Another is a protective component having an extremely negative correlation coefficient. Because rare variants have high genetic effects, they were added into the collapsed set one at a time by a weighted sum function until either there were no variants remaining, or there was no improvement of the correlation coefficient. Repeat the forward selection procedure without common variants as the basis, two more components were generated. Last, the collapsed score was obtained from the four components according to the measure of squared correlation coefficient with the trait. The test statistic then can be derived from a logistic regression model between the trait and the collapsed score as before. P-values can be computed by permutation.

Now, we describe the procedure in details. Assume there are *J *common variants and *K *rare variants within a certain predefined genomic region. Let $X_{j}^{C}$ and $X_{k}^{R}$ denote vectors across all samples for common and rare variants, defined by a threshold MAF = 0.01, where *j *= 1, . . . , *J*, and *k *= 1, . . . , *K*. Let *S*_+ _denote the deleterious component, which is a vector collapsed by the subset of the SNPs to achieve an extremely positive correlation. Let *S*_- _denote the protective component, which is a vector collapsed by the subset of the SNPs to achieve an extremely negative correlation.

Step 1: Forward selection on common SNPs with sum collapsing.

a) Calculate the correlation coefficient R for each common SNP with the trait. The common SNP with the largest correlation coefficient is added into $S_{+}^{new}$, while the common SNP with the lowest correlation coefficient is added into $S_{-}^{new}$.

$$\begin{array}{c} S_{+}^{new}=argmax_{T_{+}=collapes\left(S_{+},X_{j}^{C}\right)}\\ \left\{Cor\left(T_{+},Y\right)\text{|}Cor\left(T_{+},Y\right)>0\right\} \end{array}$$

and

$$\begin{array}{c} S_{-}^{new}=argmax_{T_{-}=collapes\left(S_{-},X_{j}^{C}\right)}\\ \left\{-Cor\left(T_{-},Y\right)\text{|}Cor\left(T_{-},Y\right)<0\right\} \end{array}$$

where $collapes\left(S_{+},X_{j}^{C}\right)$ is the sum of the vector *S*_+ _and $X_{j}^{C}$, for *j *= 1, . . . , *J*.

b) Update *S*_+ _and *S*_- _with $S_{+}^{new}$ and $S_{-}^{new}$. Let *j *take values only from the remaining common SNPs. Repeat a) until either all common variants are collapsed into components or there is no improvement for the correlation coefficient of each component.

Step 2: Forward Selection on rare SNPs with weighted sum collapsing.

a) Because rare variants have high genetic effects, the data driven weight is derived as follows to favor the rare variants with high genetic effect in both deleterious and protective way.

$$w_{k}=\frac{p_{k}}{\sum_{k}p_{k}}K$$

where $p_{k}=|\frac{\#\left\{y_{i}=1,X_{ik}^{R}>0\right\}}{\#\left\{X_{ik}^{R}>0\right\}}-0.5|$.

$X_{ik}^{R}>0$ indicates a mutation for the *i*th sample in the *k*th rare variant. *p_k _*is the empirical estimate of the probability that an individual with the mutation will have the disease. w_k _is adjusted based on p_k _with the constraint that the sum of the weights is the number of rare variants.

b) Calculate the correlation coefficient R for each rare SNP with the trait. The rare SNP with the largest correlation coefficient is added into ${\text{S}}_{+}^{\text{new}}$, while the rare SNP with the lowest correlation coefficient is added into ${\text{S}}_{-}^{\text{new}}$.

$$\begin{array}{c} S_{+}^{new}=argmax_{T_{+}=collapes\left(S_{+},X_{k}^{R}\right)}\\ \left\{Cor\left(T_{+},Y\right)\text{|}Cor\left(T_{+},Y\right)>0\right\} \end{array}$$

and

$$\begin{array}{c} S_{-}^{new}=argmax_{T_{-}=collapes\left(S_{-},X_{k}^{R}\right)}\\ \left\{-Cor\left(T_{-},Y\right)\text{|}Cor\left(T_{-},Y\right)<0\right\} \end{array}$$

where $collapes\left(S_{+},X_{k}^{R}\right)$ is the sum of the vector *S*_+ _and $w_{k}X_{k}^{R}$, for *k *= 1, . . . , *K*.

c) Update *S*_+ _and *S*_- _with $S_{+}^{new}$ and $S_{-}^{new}$. Let k take values only from the remaining rare SNPs. Repeat b) until either all rare variants are collapsed into components or there is no improvement for the correlation coefficient of each component. The whole procedure generates two collapsed scores $S_{+}^{Both}$, $S_{-}^{Both}$ representing deleterious and protective components for respectively rare variants based on common variants.

Step 3: Construct the final collapsed score. Repeat Step2 considering rare variants only without the bases from common variants. Thus, our test can be robust when common SNPs are not associated with the trait. It will generate another two components, $S_{+}^{R}$ and $S_{-}^{R}$. The final collapsed score is derived as follow.

$$S_{wSC}=argmax_{T\in A}\left\{Cor{\left(\text{T},Y\right)}^{2}\right\}$$

where $\text{A}=\left\{S_{+}^{Both},S_{-}^{Both},S_{+}^{R},S_{-}^{R}\right\}$

The test statistic (wSC) can be derived from a logistic regression model between the trait and the collapsed score as before. P-values can be computed by permutation.

S_wSC _is constructed by comparing the potential effect of components in different directions. As an alternative, we also propose a method (wSCd) to detect the genetic effects and it is robust when the effects are in different directions. To find wSCd, we will follow all the same steps described before in deriving wSC, but the final collapsed score is

$$S_{wSCd}=argmax_{T\in A}\left\{Cor{\left(\text{T},Y\right)}^{2}\right\}$$

where $\text{A}=\left\{S_{+}^{Both}-S_{-}^{Both},S_{+}^{R}-S_{-}^{R}\right\}$

## Authors' contributions

YD contributed in the development of the statistical tests, provided simulation strategies, and drafted the manuscript. RJ and JD both supervised the whole process and participated in drafting the manuscript. All authors read and approved the manuscript.

## Appendix

In the appendix, we show that the weight defined by $w_{c}=\frac{1}{\sqrt{\hat{q}\left(1-\hat{q}\right)}}$[12] tends to favor those deleterious rare variants and ignore the protective rare variants. Instead of using estimated minor allele frequencies, let q be minor allele frequency in controls, and let p be the minor allele frequency in case. Then $w=\frac{1}{\sqrt{q\left(1-q\right)}}$, and *w *and w_c _should have similar behavior.

By its definition w is a decreasing function of q, where q ∈ (0, 0.5). Let R denote the odds ratio of case and control groups and r be the minor allele frequency in all samples for a given SNP. We have

$$\text{R}=\frac{p}{1-p}/\frac{q}{1-q}$$

and

$$\frac{N_{case}p+N_{control}q}{N_{case}+N_{control}}=\text{r}$$

where *N_case_*, *N_control _*are the number of samples in cases, controls, respectively. The above equation can be written as

$$q=\text{r}-\frac{N_{case}\left(p-q\right)}{N_{case}+N_{control}}$$

The relationship between p and q can be easily derived based on the value of R as follows.

$$\begin{array}{c} \text{If}\;\text{R}>1,\frac{p}{1-p}/\frac{q}{1-q}>1\Rightarrow \text{p}>\text{q}\Rightarrow \text{q}<\text{r}.\\ \text{If}\;\text{R}=1,\frac{p}{1-p}/\frac{q}{1-q}=1\Rightarrow \text{p}>\text{q}\Rightarrow \text{q}=\text{r}.\\ \text{If}\;\text{R}<1,\frac{p}{1-p}/\frac{q}{1-q}<1\Rightarrow \text{p}<\text{q}\Rightarrow \text{q}>\text{r}. \end{array}$$

Let $w_{0}=\frac{1}{\sqrt{r\left(1-r\right)}}$, which is the weight for any non-causal variant (R = 1). If rare variants have deleterious genetic effect, then R > 1 and w > w_0_. If rare variants potentially have protective genetic effect for the disease, then R < 1 and w < w_0_. This shows that the weight defined by $w_{c}=\frac{1}{\sqrt{\hat{q}\left(1-\hat{q}\right)}}$[12] tends to favor those deleterious rare variants and ignore the protective rare variants.
